# Kafir Orange

**Published:** 1913-06

**Authors:** R. H. True

**Affiliations:** Physiologist in Charge. Washington, D. C.


**Kafir Orange.**

Washington, D. C.

Editor Buffalo Medical Journal:

Dear Sir—I beg to acknowledge the receipt of your letter relative to the Kafir orange and certain publications of the Department.

The Kafir orange (*Strychnos spinosa*) is, as you doubtless know, a native of Portuguese East Africa and has received this name from its outward resemblance to an orange. The fruit is the size of an orange, or larger, deep green when young and yellow when ripe. It has a very hard shell and numerous flat seeds imbedded in the pulp. The seeds are said to be very poisonous, but the flesh is quite refreshing. Mr. Fairchild, in charge of the office of seed and plant introduction of this bureau, who brought the seed from East Africa for trial in Florida, says of the fruit: "The specimen which we tasted was like a brandied peach into which cloves had been stuck. The spicy aroma of the fruit is perceptible before the hard shell has been broken open."

The seeds planted in Florida produced plants which flourished and developed fruit. A couple of years ago Mr. A. F. Sievers of this office made an examination of one of these Florida fruits for the alkaloids strychnine and brucine. He found neither of the alkaloids present in any part of the fruit except in the seeds, and only a very slight trace there. The fruit, however, was not fully ripe, and it is possible that the alkaloids are formed only during the final stage of ripening. The fact also that the plant from which the fruit was obtained was grown in Florida and not in Africa, where it is native, may have influenced the alkaloidal content. Very truly yours, R. H. True,

Physiologist in Charge.

